# Efficacy and safety of traditional Chinese medicine injections for older adults with community-acquired pneumonia: An evidence map

**DOI:** 10.1097/MD.0000000000046849

**Published:** 2025-12-26

**Authors:** Hao Yang, Jun Wang, Kang Zhang, Kai Xie, Wenshuai Ji, Haifeng Wang

**Affiliations:** a Department of Respiratory Medicine, The First Affiliated Hospital of Henan University of Traditional Chinese Medicine, Zhengzhou, China; b The First Clinical Medical College of Henan University of Traditional Chinese Medicine, Zhengzhou, China; c Co-construction Collaborative Innovation Center for Chinese Medicine and Respiratory Diseases by Henan and Education Ministry of P.R. China, Henan University of Chinese Medicine, Zhengzhou, China.

**Keywords:** community-acquired pneumonia, evidence map, older adults, randomized controlled trials, systematic reviews, traditional Chinese medicine injections

## Abstract

**Background:**

Community-acquired pneumonia (CAP) is a common respiratory condition associated with high morbidity and mortality. Older adults are disproportionately affected, experiencing higher risks and poorer outcomes. The current evidence regarding the efficacy and safety of traditional Chinese medicine injections (TCMIs) for CAP in older adults is limited. This study aims to synthesize high-quality evidence and identify gaps in the existing literature to guide future research.

**Methods:**

A systematic search was conducted in eight databases – PubMed, Cochrane Library, EMBASE, Web of Science, CNKI, VIP, Wanfang, and SinoMed – covering records from database inception to December 19, 2024. Randomized controlled trials (RCTs), systematic reviews, and meta-analyses (MAs) assessing the use of TCMIs for older adults with CAP were included. The risk of bias in RCTs was assessed using the Cochrane risk of bias tool. Evidence bubble plots were created to visualize the study characteristics and distribution of evidence.

**Results:**

The evidence map included 165 RCTs and one MAs, with the earliest publication dating from 2001. All studies were conducted in China, and RCTs accounted for 99% of the included evidence. Tanreqing Injection was the most frequently investigated intervention, with a median treatment duration of 7 to 13 days. The primary outcomes were the clinical effective rate and time to symptom resolution. Both the RCTs and the MAs reported significant benefits of TCMIs for older adults with CAP. However, the overall certainty of the evidence remains low.

**Conclusion:**

TCMIs show substantial potential for improving clinical efficacy and alleviating symptoms in older adults with CAP. However, existing studies have design and methodological limitations. Future research should focus on improving methodological quality to enhance the robustness of the clinical evidence.

## 1. Introduction

Community-acquired pneumonia (CAP) refers to lung parenchymal inflammation acquired outside of hospital settings.^[1]^ It is associated with high incidence, elevated mortality, and a significant disease burden, representing a major global public health concern.^[2]^ Population aging is a universal issue, with individuals aged 60 years and older currently comprising 12.3% of the global population. This proportion is projected to increase to nearly 22% by 2050.^[3]^ Due to declining organ function, multiple comorbidities, and compromised immunity, older adults are more susceptible to CAP.^[4,5]^ Furthermore, discharged older adults often experience persistent symptoms, including cough, sputum production, fatigue, and anorexia. These lingering symptoms not only severely impair the patients’ quality of life but also elevate the risk of rehospitalization and mortality.^[6]^

A systematic review estimated that approximately 6.8 million older adults are hospitalized globally each year for pneumonia, with nearly 1.1 million in-hospital deaths.^[7]^ These high hospitalization and mortality rates highlight the severity of CAP in older adults. Compared to younger adults, older adults with CAP incur higher treatment costs and experience worse clinical outcomes, placing a significant economic burden on both individuals and healthcare systems.^[8–10]^ Therefore, there is an urgent need for effective management and prevention strategies to reduce the impact of CAP on public health.

However, current therapeutic approaches for CAP have not yielded fully satisfactory outcomes. Conventional management mainly involves anti-infective therapy, general supportive care, and symptomatic treatment.^[11]^ Although antibiotic therapy remains a cornerstone of treatment, antimicrobial resistance,^[12]^ – the ability of bacteria to withstand the effects of antimicrobial agents – has become increasingly prevalent due to bacterial mutations and the misuse of antimicrobial drugs, leading to therapeutic challenges.^[13–15]^

In Eastern countries, Traditional Chinese Medicine (TCM) is commonly used as a complementary and alternative approach to conventional treatments.^[16–18]^ Studies have shown that TCM offers unique benefits in managing CAP, with lower risks of antimicrobial resistance development.^[19–21]^ Traditional Chinese Medicine Injections (TCMIs) represent a modernized pharmaceutical form that goes beyond traditional administration methods. Characterized by high bioavailability and rapid efficacy, TCMIs are one of the most widely used dosage forms of Chinese medicinal products.^[22]^ They are extensively employed in treating acute and severe conditions, including respiratory diseases like pneumonia, with demonstrated clinical efficacy, highlighting their broad applicability.^[23–25]^ Despite this, a significant gap exists: no comprehensive evidence mapping study has yet synthesized existing clinical data to inform the development of optimized CAP therapeutics.

Evidence mapping is an innovative tool for integrating evidence.^[26]^ Through comprehensive literature retrieval, it systematically synthesizes, categorizes, and analyzes existing research within a specific field, utilizing graphical and tabular presentations to visually represent the complete evidence landscape. In this study, we systematically retrieved and synthesized published clinical studies on the use of TCMIs for treating CAP in older adults. By employing the evidence mapping approach, this study provides a comprehensive evaluation of the current state and limitations of the evidence in this area, thereby offering an evidence-based foundation for clinical decision-making and serving as a reference for optimizing research protocols and developing clinical guidelines.

## 2. Methods

### 2.1. Inclusion criteria

The inclusion criteria encompassed all clinical evidence related to the use of TCMIs for treating CAP in older adults. Eligible participants were those with a confirmed diagnosis of CAP, according to internationally recognized diagnostic criteria, with no age restrictions other than being ≥ 65 years. There were no limitations based on gender, ethnicity, or geographic location. The eligible study designs included randomized controlled trials (RCTs), systematic reviews, and meta-analyses (MAs). The experimental intervention involved TCMIs in combination with conventional Western pharmacotherapy, while the control groups received either conventional Western pharmacotherapy alone, placebo, or no treatment. There were no restrictions regarding administration routes, dosage regimens, treatment duration, or publication language.

### 2.2. Exclusion criteria

The exclusion criteria were as follows: literature not relevant to the topic; animal studies, reviews, and conference abstracts; MAs that included non-randomized controlled trials, cohort studies, or case-control studies; duplicate publications; articles with unavailable full texts; studies with incomplete data; and research that excluded patients with COVID-19 to maintain homogeneity.

### 2.3. Search strategy

A comprehensive literature search was conducted to identify studies on the treatment of non-severe CAP using TCMIs. Eight databases were searched: China National Knowledge Infrastructure (CNKI), Wanfang Data, VIP Information, China Biomedical Literature Service System (SinoMed), PubMed, Embase, Web of Science, and the Cochrane Library. The search covered records from the inception of each database through December 19, 2024.

The search terms included “Pneumonia,” “Experimental Lung Inflammation,” “Lobar Pneumonia,” “Pulmonary Inflammation,” “Traditional Chinese Medicine Injection,” “Injection of Chinese Medicine,” “Tanreqing Injection,” and “Reduning Injection,” among others. The search strategy combined subject headings and free-text terms, adapted to the characteristics of each database.

### 2.4. Study selection and data extraction

The literature screening and management were carried out using EndNote X9. Two researchers independently performed an initial screening of the titles and abstracts of the retrieved articles, followed by cross-validation. Subsequently, the full texts of potentially eligible studies were reviewed. All studies meeting the predefined inclusion and exclusion criteria were identified, and relevant data were independently extracted by the two researchers using Microsoft Excel 2021. The extracted information included the title, authors, year of publication, province, funding source, study design, sample size, duration of treatment, interventions, and outcome measures. After data extraction, cross-checking was conducted to ensure the accuracy and completeness of the information. Any discrepancies were resolved through group discussions.

### 2.5. Quality assessment

Two trained and qualified researchers independently assessed the risk of bias in the included RCTs. In cases of disagreement, a third researcher was consulted to resolve discrepancies and determine the final assessment.

The Cochrane risk of bias (ROB) tool was used to evaluate the risk of bias in the included RCTs.^[27]^ The assessment covers seven domains: sequence generation of randomization, allocation concealment, blinding of participants and personnel, blinding of outcome assessors, completeness of outcome data, selective reporting, and other biases. Each domain was classified as “high risk,” “low risk,” or “unclear risk.”

### 2.6. Statistical analysis

The evidence was presented through a combination of figures, tables, and descriptive text. Data were summarized using frequency counts and percentages. The distribution characteristics of the studies and evidence were illustrated using line charts, bar charts, bubble charts, and pie charts.

## 3. Results

### 3.1. Selection of sources of evidence

**Figure 1.**
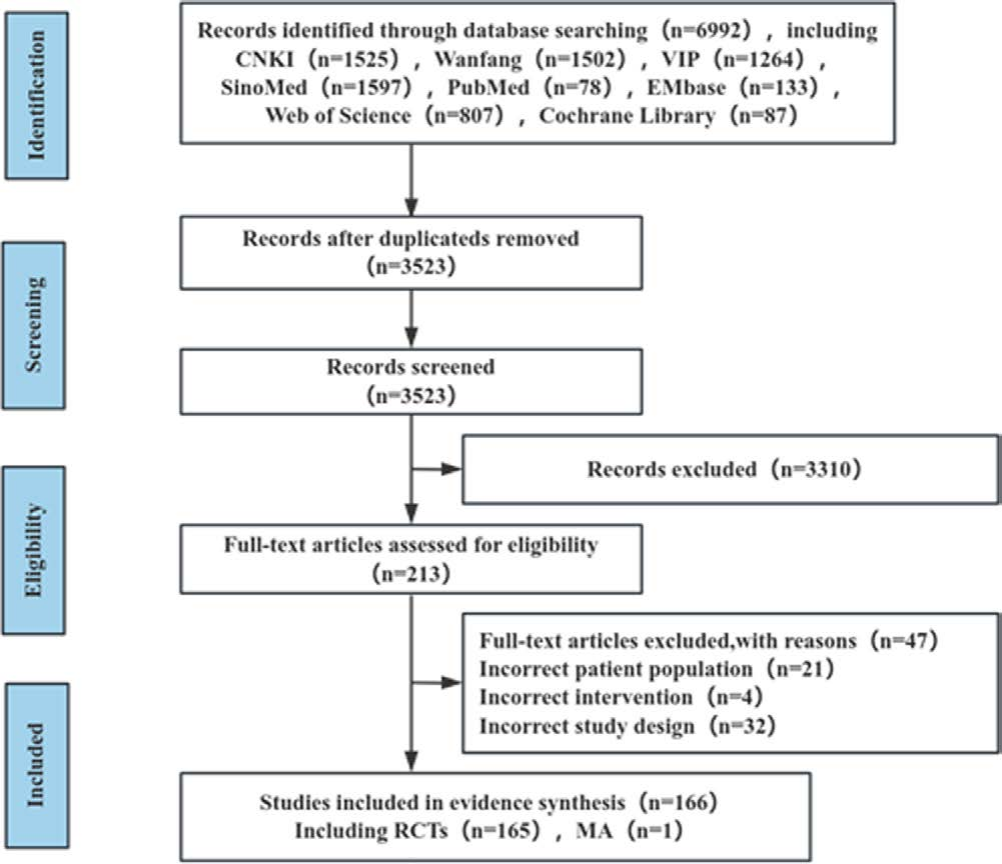
Flowchart of the literature review process and results. MAs = meta-analyses, RCTs = randomized controlled trials.

A total of 6992 records were identified through database searches. After removing 3469 duplicates, 3310 publications were excluded during title and abstract screening due to non-relevance. The remaining 213 full-text articles were assessed for eligibility. Of these, 21 were excluded for ineligible population characteristics, 4 for incorrect interventions, and 22 for non-compliant study designs. Ultimately, 166 publications were included, comprising 165 RCTs and 1 MAs. The complete screening workflow is shown in Figure 1.

### 3.2. Study characteristics

#### 3.2.1. Annual trends in publications

**Figure 2.**
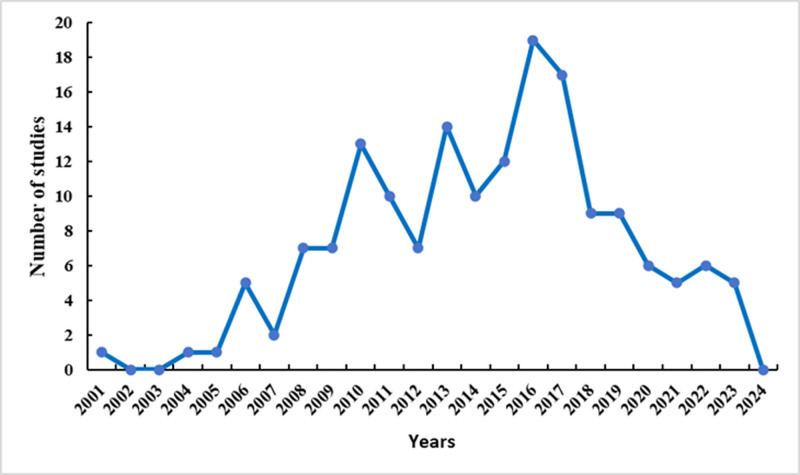
Annual publication volume of traditional Chinese medicine injections for older adult community-acquired pneumonia.

To visualize the temporal trends of publications on TCMIs for treating older adult CAP, a chronological distribution analysis was conducted. The included publications spanned from 2001 to 2024, with RCTs first appearing in 2001 and the first MAs published in 2018. The research trajectory showed that the annual publication volume fluctuated, with an overall upward trend since 2001, peaking in 2016. Since then, publications on TCMIs for older adult CAP have followed a fluctuating downward trend. These temporal trends are visualized in Figure 2.

#### 3.2.2. Geographical distribution

**Figure 3.**
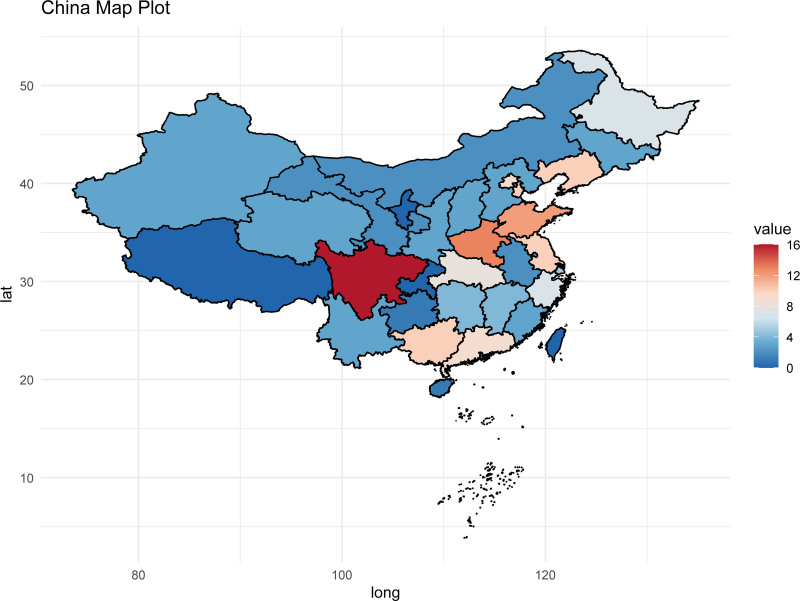
Geographic distribution of principal investigators in studies on traditional Chinese medicine injectable preparations for older adult community-acquired pneumonia.

All 166 included studies were conducted exclusively in mainland China, distributed across 28 provincial-level administrative regions. Sichuan Province had the highest number of publications (n = 16), followed by Henan Province (n = 13), Shandong Province (n = 12), and Guangxi Zhuang Autonomous Region (n = 10). No eligible publications were found in the Tibet Autonomous Region, Ningxia Hui Autonomous Region, Chongqing Municipality, Hong Kong Special Administrative Region, Macao Special Administrative Region, or Taiwan. The geographical distribution is shown in Figure 3. The color gradient transitions from blue to red, representing the variation in the number of studies. Red regions indicate a higher number of studies, while blue regions suggest a lower number or lack of relevant research.

#### 3.2.3. Funding sources

Among the 166 included studies, 10 (6.02%) explicitly documented funding support. Of these, 1 (0.6%) study was funded by a national-level grant, while 9 (5.42%) studies received funding from provincial or lower-level grants. The funded studies included 9 (5.42%) RCTs and 1 (0.6%) MAs. The remaining 156 studies (93.98%) did not receive any funding support.

### 3.3. The basic characteristics of RCTs

#### 3.3.1. Types of traditional Chinese medicine injections

**Figure 4.**
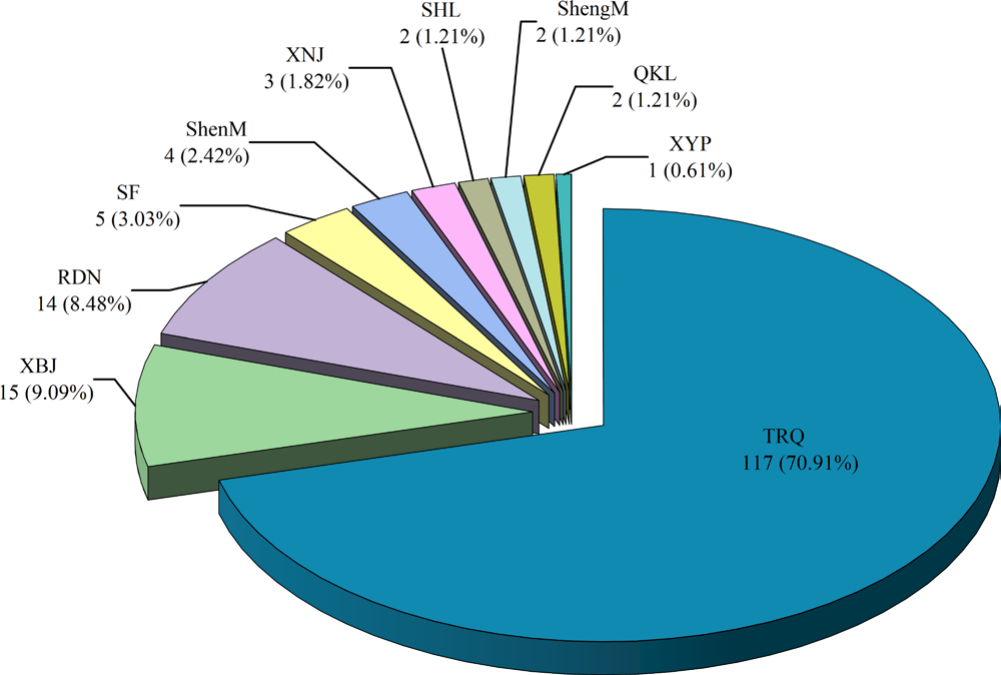
Proportions of various traditional Chinese medicine injections.

Among the 165 included RCTs, 10 types of TCMIs were identified. Tanreqing injection had the highest number of publications (n = 117, 70.9%), followed by Xuebijing Injection (n = 15, 9.1%) and Reduning Injection (n = 14, 8.5%), as shown in Figure 4. The botanical nomenclature of the herbal components in these injections was standardized according to the Pharmacopoeia of the People’s Republic of China (2020 Edition; available at https://ydz.chp.org.cn/). The profiles of the TCMIs are summarized in Table 1.

**Table 1:** Summary of included 10 traditional Chinese medicine injections.

Medicine name	Abbreviation	Composition
Tanreqing Inj	TRQ	Scutellaria baicalensis Georgi, Bear Gall, Goral Horn, Lonicera japonica Thunb, Lonicera japonica Thunb, Forsythia suspensa (Thunb.) vahl
Xuebijing Inj	XBJ	Carthamus tinctorius L, Paeonia lactiflora Pall, Ligusticum chuanxiong Hort, Salvia miltiorrhiza Bge, Angelica sinensis (Oliv.) Diels
Reduning Inj	RDN	Artemisia annua L, Lonicera japonica Thunb, Gardenia jasminoides Ellis
Shenfu Inj	SF	Panax ginseng C.A.Mey, Aconitum carmichaelii Debx
Shenmai Inj	ShenM	Panax ginseng C.A.Mey, Ophiopogon japonicus (L.f) Ker-Gawl
Xingnaojing Inj	XNJ	Moschus berezovskii Flerov, Gardenia jasminoides Ellisi, Curcuma wenyujin Y.H.Chen et C.Ling, Borneolum Syntheticum
Shuanghuanglian Inj	SHL	Lonicera japonica Thunb, Scutellaria baicalensis Georgi, Forsythia suspensa (Thunb.) Vahl
Shengmai Inj	ShengM	Panax ginseng C.A.Mey, Ophiopogon japonicus (L.f) Ker-Gawl, Schisandra chinensis (Turcz.) Baill
Qingkailing Inj	QKL	Cholic Acid, Hyriopsis cumingii (Lea), Deoxycholic Acid, Gardenia jasminoides Ellis, *Bubali Cornu*, lsatis indigotica Fort, Baicalin, Lonicera japonica Thunb
Xiyanping Inj	XYP	Total Sulfonated Andrographolide

#### 3.3.2. Study settings and sample sizes

**Figure 5.**
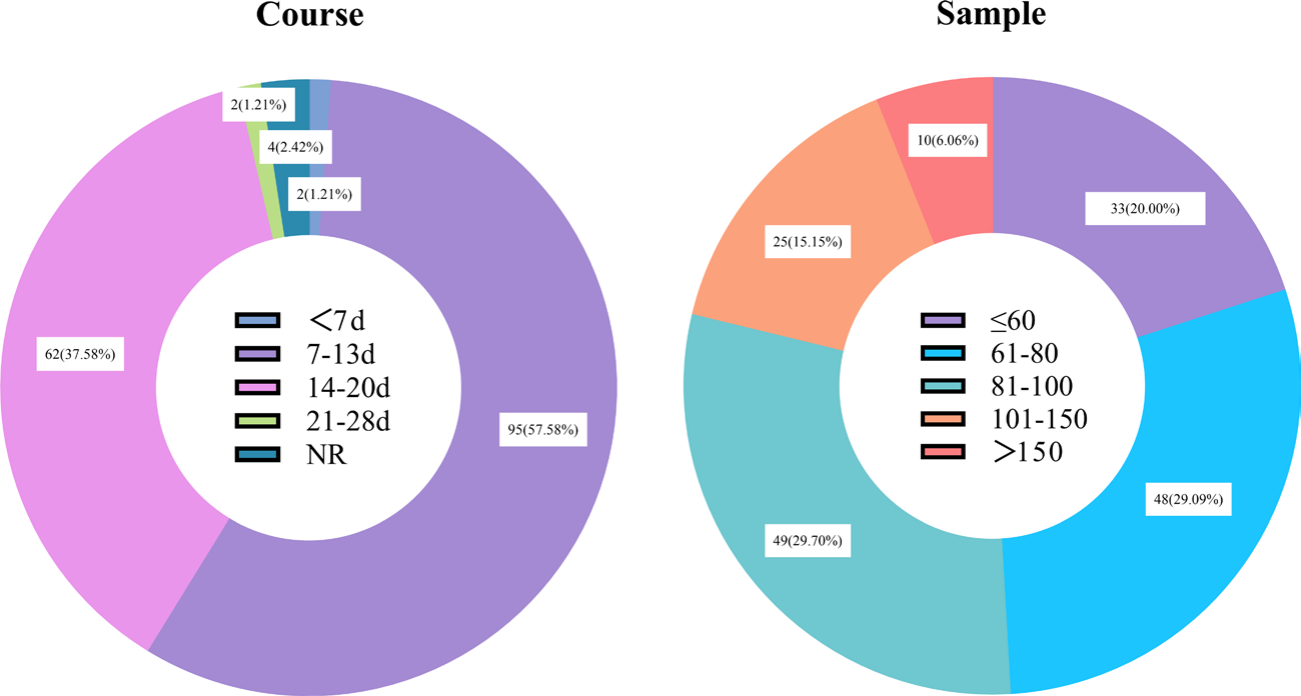
Duration and sample size distribution of randomized controlled trials of traditional Chinese medicine injections for the treatment of community-acquired pneumonia in older adults.

Among the 165 RCTs, 163 (98.8%) were conducted at single centers, while 2 (1.2%) did not specify their study settings. No multicenter trials were identified. These studies collectively enrolled 14,755 participants, with sample sizes ranging from 31 to 295 participants. The majority of trials (n = 130, 78.8%) had sample sizes of 100 participants or fewer, 25 trials (15.2%) enrolled 101–150 participants, and only 10 trials (6.0%) had sample sizes exceeding 150 participants. The distribution of sample sizes is shown in Figure 5A.

#### 3.3.3. Course of treatment

Among the 165 RCTs, 161 (97.6%) explicitly documented the treatment duration. Treatment durations ranged from 5 to 28 days, with the majority of trials concentrated in two intervals: 7–13 days (n = 95, 57.6%) and 14–20 days (n = 62, 37.6%). The distribution of treatment durations for TCMIs in older adult CAP is shown in Figure 5B.

#### 3.3.4. TCM syndrome

Among the 165 included RCTs, 18 (10.91%) reported TCM syndrome patterns in older adult CAP. The documented syndromes included: Phlegm-heat obstructing the lung syndrome (n = 14, 8.48%), Wind-warmth lung heat syndrome (n = 2, 1.21%), Wind-heat invading the lung syndrome (n = 3, 1.82%), Qi-yin deficiency syndrome (n = 1, 0.61%), and Deficiency-rooted lingering pathogen syndrome (n = 1, 0.61%). Notably, one RCT reported two concurrent syndromes, while another reported three coexisting patterns.

### 3.4. Quality assessment

#### 3.4.1. Risk of bias for the 486 included RCTs

**Figure 6.**
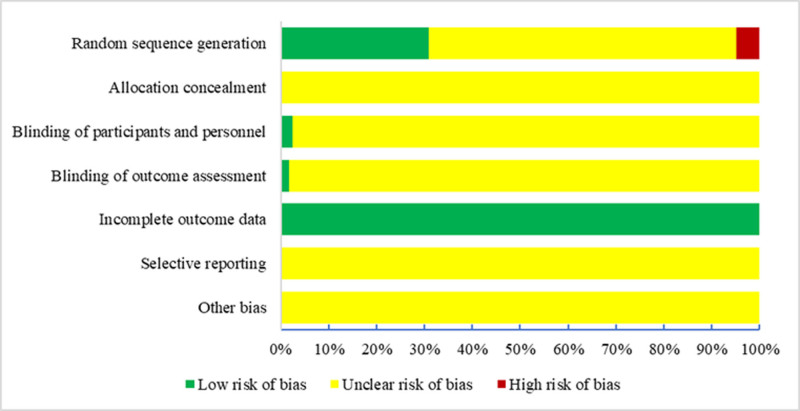
Evaluation of methodological quality for randomized controlled trials.

The Cochrane ROB tool was used to assess the risk of bias in all 165 included RCTs. The results are as follows: (1) 51 RCTs (30.9%) used rigorous randomization methods (e.g., random number tables, computer-generated randomization, and lottery) and were rated as “low risk”; 8 RCTs (4.8%) used quasi-randomization methods (e.g., randomization based on admission sequence or treatment preference) and were rated as “high risk”; 106 RCTs (64.2%) mentioned “randomization” but did not specify the method and were rated as “unclear risk.” (2) Allocation concealment: No RCTs described appropriate allocation concealment methods and were rated as “unclear risk.” (3) Blinding of participants and personnel: 4 RCTs (2.4%) implemented blinding for participants and personnel and were rated as “low risk”; 161 RCTs (97.6%) did not mention blinding and were rated as “unclear risk.” (4) Blinding of outcome assessors: 3 RCTs (1.8%) implemented blinding for outcome assessors and were rated as “low risk”; the remaining studies were rated as “unclear risk.” (5) Incomplete outcome data: All RCTs (100%) had no missing data or missing data that did not affect the analysis and were rated as “low risk.” (6) Selective reporting: None of the 165 RCTs mentioned protocol registration and were rated as “unclear risk.” (7) Other biases: It was not possible to determine the presence of other biases in the 165 RCTs, which were rated as “unclear risk.” The risk of bias profile is presented in Figure 6.

#### 3.4.2. Outcome Measures

A total of 165 RCTs on TCMIs for treating older adult CAP reported outcomes across seven primary categories: (1) Short-term efficacy: Evaluated through clinical response rate, mortality, length of hospital stay, and bacterial clearance rate. (2) Long-term prognosis: Assessed via recurrence rate. (3) Clinical signs and symptoms: Measured through the time to improvement of clinical manifestations, clinical symptom and sign scores, and TCM syndrome scores. (4) Laboratory parameters: Including pulmonary function tests (e.g., diffusing capacity for carbon monoxide, total lung capacity, forced expiratory volume in 1 second, forced vital capacity), arterial blood gas analysis (e.g., partial pressure of oxygen [PaO₂], partial pressure of carbon dioxide [PaCO₂], pH), coagulation function, pulmonary imaging examinations, inflammatory markers, and immune function indices. (5) Quality of life: Measured using the Clinical Pulmonary Infection Score and Functional Independence Measure. (6) Safety outcomes: Documenting adverse events. (7) Other endpoints: Covering antibiotic utilization rates.

**Figure 7.**
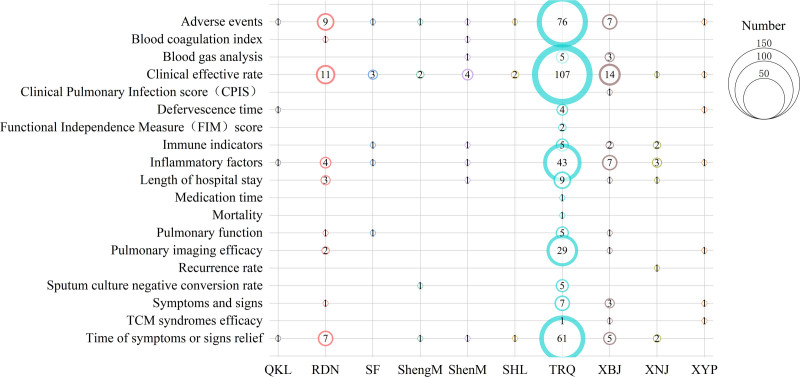
Distribution of outcome measures in RCTs on traditional Chinese medicine injections for older adult community-acquired pneumonia.

Overall, safety outcomes and certain core efficacy endpoints—such as clinical response rate and time to improvement of clinical manifestations—received more research attention, while investigations into long-term prognosis, quality of life assessments, and health economic indicators were relatively limited. The bubble diagram illustrating outcome measures for TCMIs in treating older adult CAP is presented in Figure 7: Distribution of Outcome Measures in RCTs on TCMIs for Older Adult CAP. The size of the bubbles represents the number of included RCTs. Different colors represent various interventions, and the horizontal axis shows various intervention categories, while the vertical axis represents efficacy outcomes.

### 3.5. Specific findings from MAs in the evidence mapping

Only one MAs on TCMIs for treating older adult CAP was included, encompassing 23 RCTs with a total of 2207 participants. The results demonstrated that Tanreqing Injection (TRQ) significantly improved the overall response rate, shortened the time to improvement of clinical manifestations, reduced inflammatory markers, and exhibited a favorable safety profile in older adult CAP patients.

## 4. Discussion

### 4.1. Summary of the main findings

This study systematically retrieved literature on RCTs and systematic reviews/MAs of TCMIs for treating older adult CAP published from database inception to December 19, 2024. A total of 166 studies were included. Evidence mapping methodology was employed to systematically collate 165 RCTs and 1 meta-analysis. The Cochrane ROB tool was used to assess the risk of bias in the included RCTs. This study objectively presents the current research landscape and evidence distribution concerning TCMIs for treating older adult CAP.

### 4.2. General Information

The publication trend of clinical evidence on TCMIs for treating older adult CAP has shown a fluctuating upward trajectory since 2001, peaking in 2016, followed by a fluctuating decline. This pattern may be attributed to stricter national drug regulatory policies, particularly the 2017 issuance of the “Opinions on Deepening the Reform of Review and Approval Systems to Encourage Innovation in Drugs and Medical Devices,” which imposed tighter controls on TCMIs.^[28]^ All studies were conducted exclusively in China, with concentrations in provinces such as Sichuan, Henan, and Shandong, revealing significant regional disparities. Notably, all studies were single-center studies, highlighting the urgent need for multicenter, cross-regional collaborative high-quality research. Furthermore, only 10 (6.02%) of the included studies received government funding. We hypothesize that the lack of multicenter collaboration and cross-regional studies may be related to insufficient research funding. There is a critical need for substantial future investment to support multicenter, high-quality studies across regions, thereby enhancing the evidence base for TCMIs in treating older adult CAP.

### 4.3. Study design

RCTs represent the gold standard for evaluating intervention efficacy and safety, providing the most definitive evidence on treatment outcomes.^[29]^ In this study, 99% of the evidence originated from RCTs, with sample sizes predominantly ranging from 61 to 100 participants. Most RCTs had relatively small sample sizes. Regarding treatment duration, the intervention periods were varied and lacked unified standards. The absence of follow-up in these studies precluded the assessment of the long-term effects of TCMIs in treating older adult CAP, potentially leading to an underestimation of clinical efficacy. Studies indicate that older adult CAP patients face high readmission risks post-discharge. For instance, a Spanish study involving 1756 older adult CAP inpatients across 20 hospitals found that 200 patients (11.39%) were readmitted within 30 days post-discharge.^[30]^ This underscores the need for enhanced post-discharge follow-up to evaluate long-term outcomes.

The primary TCMIs used was TRQ, which accounted for 70.91% of the studies. TRQ demonstrates potent antimicrobial activity, with documented efficacy against influenza virus, adenovirus, respiratory syncytial virus, and coxsackievirus, as well as inhibitory effects on *Streptococcus pneumoniae, Staphylococcus aureus, β-hemolytic streptococci, Haemophilus influenzae,* and *Legionella* species.^[31,32]^ According to TCM theory, TRQ clears heat, resolves toxicity, and dispels phlegm, and is widely used in China for respiratory infections and pneumonia associated with phlegm-heat obstructing the lung syndrome.^[33–35]^ Syndrome differentiation is the core of TCM practice, where diagnostic accuracy determines clinical efficacy. Notably, only 18 RCTs (10.91%) among the 165 included studies reported TCM syndrome patterns, with 14 (8.48%) specifying phlegm-heat obstructing the lung syndrome. This highlights a critical gap in attention to TCM syndrome characterization in RCTs of TCMIs for older adult CAP.

### 4.4. Study quality

RCTs are considered the gold standard for evaluating the effects of clinical interventions. However, the overall quality of RCTs on TCMIs for treating older adult patients with CAP is suboptimal, which may undermine the credibility of clinical efficacy findings.^[36]^ The included studies commonly lacked trial protocol registration and sample size estimation, which may lead to selective outcome reporting. Most studies did not implement appropriate randomization methods or blinding, and there was no mention of allocation concealment or conflicts of interest. As a result, the majority of risk-of-bias items were rated as “unclear risk,” compromising the authenticity of the research outcomes and the reliability of the evidence. Quality improvement is crucial. Future studies should refine research protocols, register them in advance, adhere to reporting standards, and ensure complete documentation of study details prior to publication.

### 4.5. Outcome measures

Outcome measures are crucial for evaluating the effectiveness of interventions. Their objectivity and appropriateness are not only closely related to assessing therapeutic efficacy but also impact clinical decision-making.^[37]^ Through comprehensive analysis, several issues were identified in the outcome measures for TCMIs in treating older adult CAP: Lack of primary outcome specification and distinction between primary and secondary outcomes: Primary outcomes are the ultimate indicators of therapeutic efficacy and best reflect the potency of interventions in clinical research.^[38]^ Among the 165 included RCTs, most failed to clearly define primary versus secondary outcomes. This omission directly impacted study design, sample size estimation, and patient benefit considerations. Neglect of quality-of-life assessment and long-term follow-up: With the increasing adoption of the biopsychosocial model in modern medicine, quality-of-life measures have gained prominence as potential primary endpoints for prognostic evaluation. For example, Steel JL et al prospectively demonstrated the prognostic value of health-related quality of life in hepatocellular and cholangiocarcinoma, suggesting its utility in patient stratification.^[39]^ However, only 3 RCTs in our review incorporated quality-of-life scales, and none included follow-up protocols. Insufficient attention to health economic indicators: TCM offers cost-saving advantages and is a cost-effective treatment option.^[40]^ Economic evaluation metrics should therefore be essential outcome measures in trials of older adult CAP. Yet, none of the included RCTs reported such data. Heterogeneity and non-standardization of TCM syndrome patterns: Syndrome differentiation, the core of Traditional Chinese Medicine diagnosis and treatment, involves identifying and treating patterns of disharmony in the body.^[41]^ Among the 165 RCTs, only 18 (10.91%) reported TCM syndrome patterns, with inconsistent terminology and diagnostic criteria for identical patterns.

To improve the quality of clinical research on TCMIs for older adult CAP, future clinical trials should adopt standardized disease and TCM syndrome reference criteria, define primary outcomes aligned with study objectives, distinguish between primary and secondary outcomes, incorporate health economic evaluations and quality-of-life assessments, and implement structured follow-up protocols. These improvements will contribute to more robust and reliable clinical evidence, ultimately enhancing patient care and outcomes.

### 4.6. Strengths and limitations

This study is the first to apply evidence mapping to elucidate and visualize the current landscape of TCMIs for treating older adult CAP. However, several limitations should be acknowledged: We only searched eight commonly used Chinese and English databases and did not include gray literature or documents from other sources, such as clinical trial registries, which may introduce bias. All studies were conducted in China, which may limit the generalizability of the findings. Further research is needed to apply these results to other countries and populations. Only one MAs was included, leading to evidence fragmentation and affecting the comprehensive assessment of the quality of the evidence.

In the future, more rigorously designed multicenter, large-sample RCTs and high-quality meta-analyses should be conducted to strengthen the evidence base for the treatment of older adult CAP with TCMIs, providing a robust foundation for their clinical application.

## 5. Conclusion

In summary, current RCTs in this field exhibit several limitations, including inadequate study design, limited cross-regional collaboration, insufficient long-term follow-up, and non-standardized outcome measures. As a result, future research should focus on multicenter, large-sample clinical trials with methodologically rigorous approaches. The literature included in meta-analyses demonstrates suboptimal quality and limited quantity. Overall, TCMIs show certain therapeutic advantages for older adult CAP. However, more stringent methodological designs and higher-quality clinical trials are needed to guide future clinical practice.

## Acknowledgments

The authors sincerely thank the faculty and students of Henan University of Traditional Chinese Medicine and the First Affiliated Hospital of Henan University of Traditional Chinese Medicine for their assistance.

## Author contributions

**Conceptualization:** Hao Yang, Haifeng Wang.

**Data curation:** Hao Yang, Kang Zhang, Kai Xie, Wenshuai Ji.

**Formal analysis:** Hao Yang, Kang Zhang, Wenshuai Ji.

**Funding acquisition:** Haifeng Wang.

**Investigation:** Hao Yang, Kang Zhang, Wenshuai Ji.

**Methodology:** Hao Yang, Jun Wang, Kang Zhang, Wenshuai Ji.

**Resources:** Haifeng Wang.

**Software:** Hao Yang, Jun Wang, Kai Xie.

**Supervision:** Haifeng Wang.

**Validation:** Kai Xie, Haifeng Wang.

**Visualization:** Jun Wang, Kai Xie, Haifeng Wang.

**Writing – original draft:** Hao Yang.

**Writing – review & editing:** Hao Yang, Haifeng Wang.
